# Supplementary material for: Decontamination of N95 and surgical masks using a treatment based on a continuous gas phase-Advanced Oxidation Process
Source: PLoS One. 2021 Mar 18;16(3):e0248487. doi: 10.1371/journal.pone.0248487 (PMC7971510; doi:10.1371/journal.pone.0248487)
Supplement: S1 Fig — The masks were loaded onto holders then passed through a hydrogen peroxide mist with ozone gas being introduced via side vents. The masks then pass under UV-C lamps with a total transit time of 30s. The flow rate of hydrogen peroxide, ozone concentration and UV-C intensity monitored via sensors. (DOCX) [file pone.0248487.s001.docx]

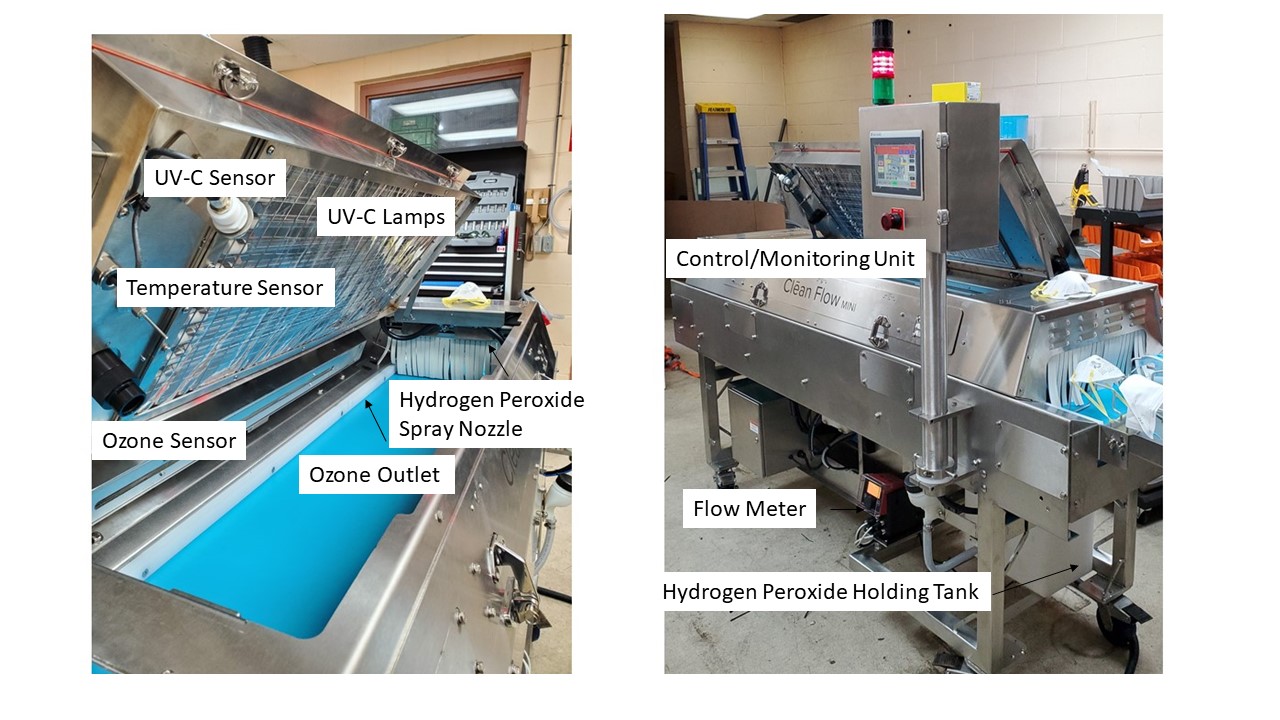


Figure 1S: Gas-Phase Advanced Oxidation Process unit for N95 and surgical mask decontamination. The masks were loaded onto holders then passed through a hydrogen peroxide mist with ozone gas being introduced via side vents. The masks then pass under UV-C lamps with a total transit time of 30s. The flow rate of hydrogen peroxide, ozone concentration and UV-C intensity monitored via sensors.
