# Supplementary material for: Decontamination of N95 and surgical masks using a treatment based on a continuous gas phase-Advanced Oxidation Process
Source: PLoS One. 2021 Mar 18;16(3):e0248487. doi: 10.1371/journal.pone.0248487 (PMC7971510; doi:10.1371/journal.pone.0248487)
Supplement: S2 Fig — Treatment 1 was when the masks were placed down for the first pass then inverted for the second pass. Treatment 2 and 3 was when the N95 masks were held vertical and Treatment 4 and 5 when held horizontally during passage through the reactor. (DOCX) [file pone.0248487.s002.docx]

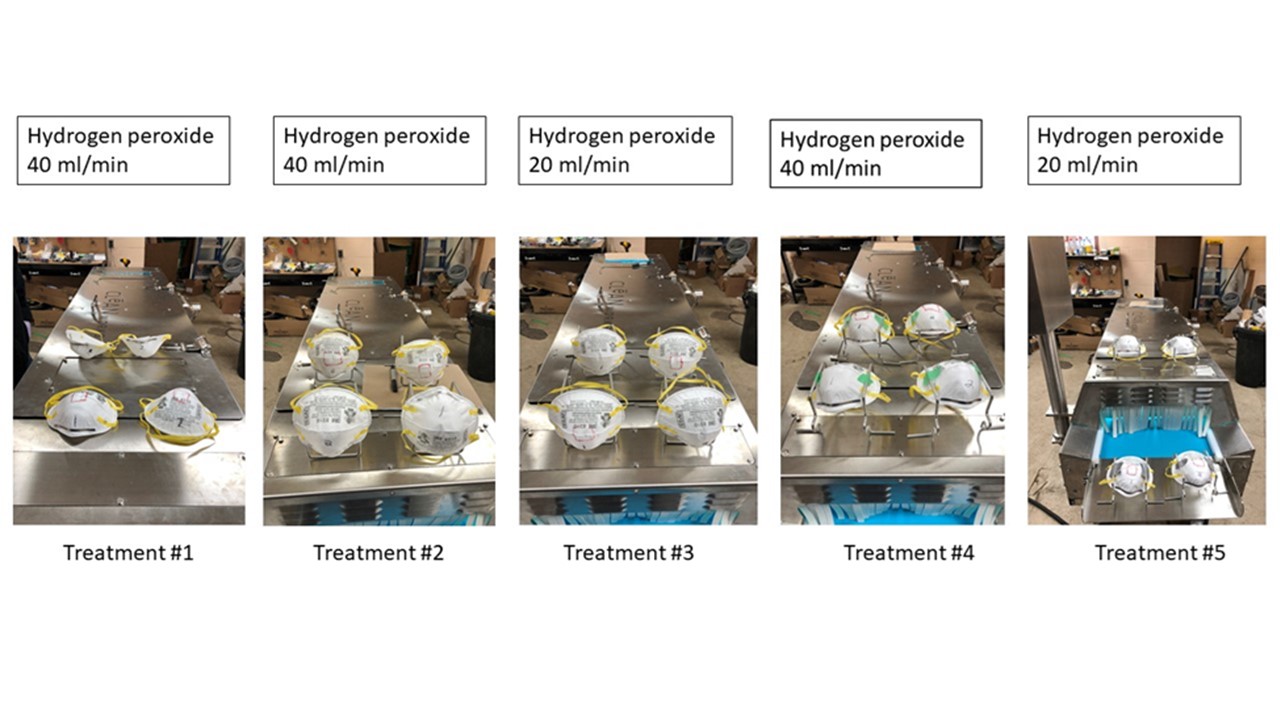


Figure 2S: Orientation of N95 masks inoculated with *Escherichia coli* K12 during passage through the gas phase Advanced Oxidation Process unit. Treatment 1 was when the masks were placed down for the first pass then inverted for the second pass. Treatment 2 and 3 was when the N95 masks were held vertical and Treatment 4 and 5 when held horizontally during passage through the reactor.
