# Supplementary material for: Decontamination of N95 and surgical masks using a treatment based on a continuous gas phase-Advanced Oxidation Process
Source: PLoS One. 2021 Mar 18;16(3):e0248487. doi: 10.1371/journal.pone.0248487 (PMC7971510; doi:10.1371/journal.pone.0248487)
Supplement: S3 Fig — The Geobacillus endospores (0.1 ml of 7 log CFU/ml) was inoculated into the marked areas on the outside (A, B, C) or inside (D, E, F) of surgical masks then allowed to dry for at least one hour before treated with the gAOP reactor. (DOCX) [file pone.0248487.s003.docx]

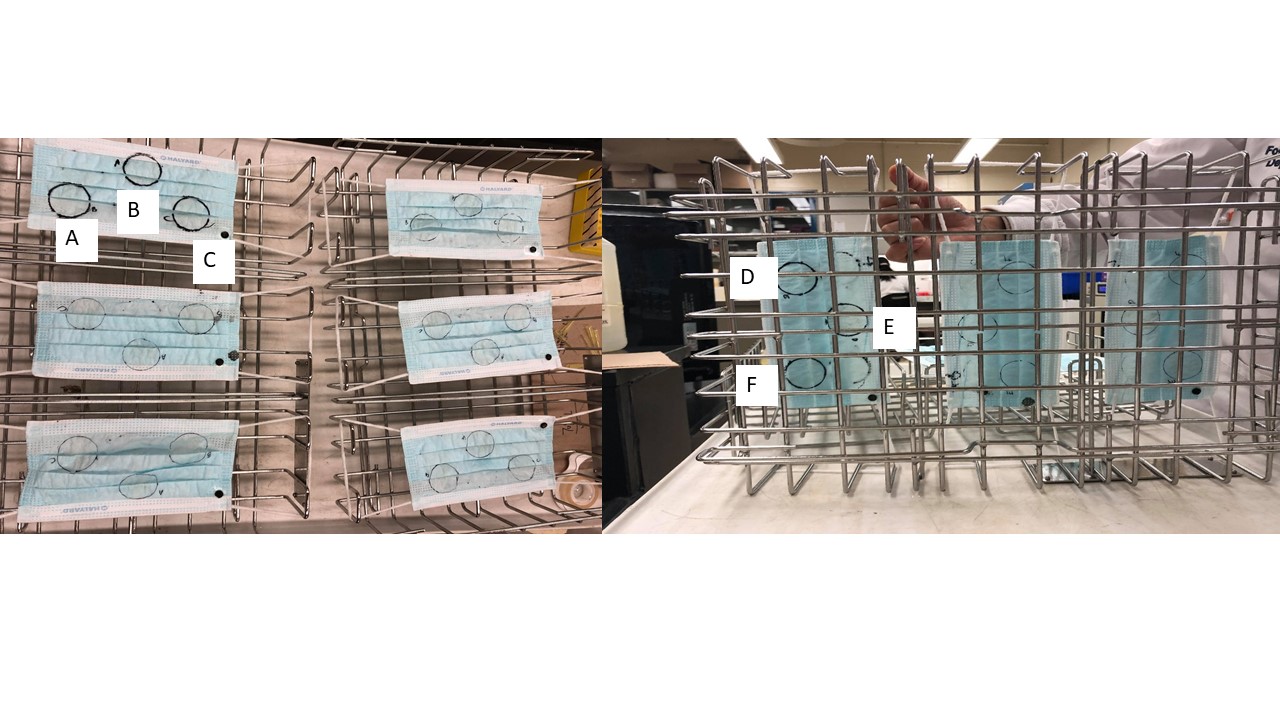


Figure 3S: Surgical masks inoculated with *Geobacillus stearothermophilus* endospores prior to passing through the gas phase Advanced Oxidation Process reactor. The *Geobacillus* endospores (0.1 ml of 7 log CFU/ml) was inoculated into the marked areas on the outside (A, B, C) or inside (D, E, F) of surgical masks then allowed to dry for at least one hour before treated with the gAOP reactor.
