# Supplementary material for: Decontamination of N95 and surgical masks using a treatment based on a continuous gas phase-Advanced Oxidation Process
Source: PLoS One. 2021 Mar 18;16(3):e0248487. doi: 10.1371/journal.pone.0248487 (PMC7971510; doi:10.1371/journal.pone.0248487)
Supplement: S4 Fig — In each configuration the N95 masks were run through the Clean Flow unit positioned face-up. (DOCX) [file pone.0248487.s004.docx]

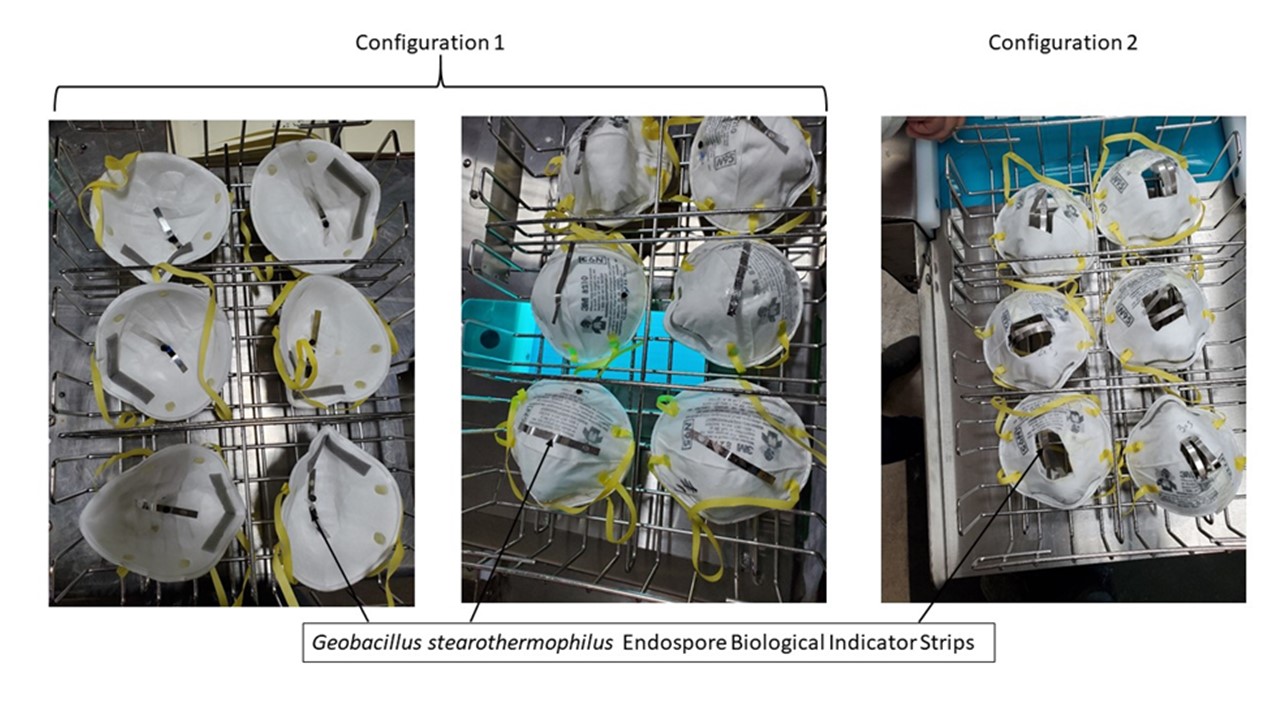


Figure 4S: 1 Biological (*Geobacillus stearothermophilus*) indicator strips positioned on the exterior or interior of N95 masks (Configuration 1) or bridged across a cut out section (Configuration 2). In each configuration the N95 masks were run through the Clean Flow unit positioned face-up.
