# Supplementary material for: Decontamination of N95 and surgical masks using a treatment based on a continuous gas phase-Advanced Oxidation Process
Source: PLoS One. 2021 Mar 18;16(3):e0248487. doi: 10.1371/journal.pone.0248487 (PMC7971510; doi:10.1371/journal.pone.0248487)
Supplement: S5 Fig — (DOCX) [file pone.0248487.s005.docx]

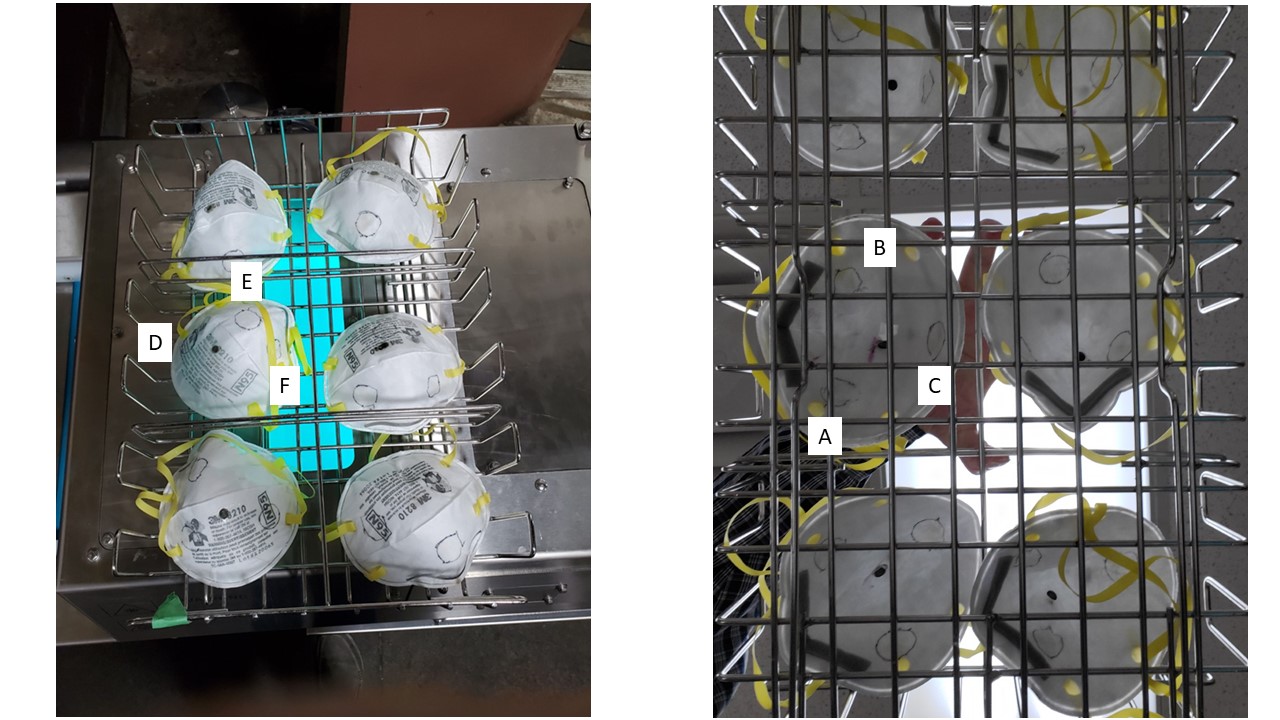


Figure 5S: N95 masks inoculated onto the marked areas on the interior (A, B, C) or exterior (D, E, F) with *Geobacillus* spores (0.1 ml of 7 log CFU/ml) then passed through the gas phase Advanced Oxidation Process reactor.
