# Supplementary material for: Decontamination of N95 and surgical masks using a treatment based on a continuous gas phase-Advanced Oxidation Process
Source: PLoS One. 2021 Mar 18;16(3):e0248487. doi: 10.1371/journal.pone.0248487 (PMC7971510; doi:10.1371/journal.pone.0248487)
Supplement: S1 Table — The masks were passed through the gAOP unit flat, horizontally or vertically as depicted in S2 Fig. (DOCX) [file pone.0248487.s006.docx]

Table S1: Log count reduction of *Escherichia coli* K12 inoculated onto the interior or exterior of N95 masks then passed through the gas phase Advanced Oxidation Process unit operating at different hydrogen peroxide flow rates. The masks were passed through the gAOP unit flat, horizontally or vertically as depicted in S2 Fig

| Treatment | Inoculation site/Mask | Log CFU | LCR | Average LCR |
| --- | --- | --- | --- | --- |
| Control | Outside 1 | 7.69 | Outside | 7.60±0.12 |
|  | Inside 1 | 4.96 | Inside | 4.86±0.14 |
|  | Outside 2 | 7.52 |  |  |
|  | Inside 2 | 4.77 | 5.91 | 5.91 |
| 1 | Outside 1 | <1.70 | 3.15 | 3.15 |
|  | Inside 1 | <1.70 | 5.91 |  |
|  | Outside 2 | <1.70 | 3.15 |  |
|  | Outside 2 | <1.70 | 4.48 | 4.54±0.09 |
| 2 | Outside 1 | 3.13 | 3.15 | 3.15 |
|  | Inside 1 | <1.70 | 4.61 |  |
|  | Outside 2 | 3.00 | 3.15 |  |
|  | Outside 2 | <1.70 | 4.07 | 4.11±0.05 |
| 3 | Outside 1 | 3.54 | 3.15 | 2.91±0.34 |
|  | Inside 1 | <1.70 | 4.14 |  |
|  | Outside 2 | 3.47 | 2.67 |  |
|  | Outside 2 | 2.18 | 5.91 | 5.91 |
| 4 | Outside 1 | <1.70 | 3.15 | 3.15 |
|  | Inside 1 | <1.70 | 5.91 |  |
|  | Outside 2 | <1.70 | 3.15 |  |
|  | Outside 2 | <1.70 | 2.32 | 2.34±0.03 |
| 5 | Outside 1 | 5.29 | 3.15 | 3.15 |
|  | Inside 1 | <1.70 | 2.36 |  |
|  | Outside 2 | 5.25 | 3.15 |  |
|  | Outside 2 | <1.70 | 5.91 | 5.91 |
| 6 | Outside 1 | <1.70 | 3.15 | 3.15 |
|  | Inside 1 | <1.70 | 5.91 |  |
|  | Outside 2 | <1.70 | 3.15 | 5.91 |
|  | Outside 2 | <1.70 | 5.91 | 3.15 |
