# Supplementary material for: Decontamination of N95 and surgical masks using a treatment based on a continuous gas phase-Advanced Oxidation Process
Source: PLoS One. 2021 Mar 18;16(3):e0248487. doi: 10.1371/journal.pone.0248487 (PMC7971510; doi:10.1371/journal.pone.0248487)
Supplement: S2 Table — E. coli was inoculated onto 2 cm x 2 cm sections of 3M 1820 N95 masks with samples (N = 3) taken at different times during holding at room temperature. The sections were suspended in saline and vortexed then levels of E. coli enumerated. (DOCX) [file pone.0248487.s007.docx]

Table S2: Viability of *Escherichia coli* K12 on the interior or exterior of N95 masks. *E. coli* was inoculated onto 2 cm x 2 cm sections of 3M 1820 N95 masks with samples (N=3) taken at different times during holding at room temperature. The sections were suspended in saline and vortexed then levels of *E. coli* enumerated.

| **Location on Mask/Time Point** | **Log CFU** | **Log N/No** |
| --- | --- | --- |
| Inside |  |  |
| 0 | 7.11 | 0 |
|  | 7.16 |  |
|  | 7.14 |  |
| 15 | 6.75 | -0.39 |
|  | 6.95 | -0.19 |
|  | 6.93 | -0.21 |
| 30 | 6.75 | -0.39 |
|  | 6.65 | -0.49 |
|  | 6.48 | -0.66 |
| 45 | 6.14 | -1.00 |
|  | 6.32 | -0.82 |
|  | 6.10 | -1.04 |
| 60 | 5.02 | -2.12 |
|  | 5.09 | -2.05 |
|  | 5.23 | -1.91 |
| Outside |  |  |
| 0 | 7.58 | 0 |
|  | 7.57 |  |
|  | 7.55 |  |
| 15 | 7.51 | -0.05 |
|  | 7.47 | -0.10 |
|  | 7.52 | -0.05 |
| 30 | 7.51 | -0.06 |
|  | 7.46 | -0.11 |
|  | 7.49 | -0.08 |
| 45 | 7.42 | -0.15 |
|  | 7.44 | -0.13 |
|  | 7.46 | -0.11 |
| 60 | 7.21 | -0.36 |
|  | 7.28 | -0.29 |
|  | 7.18 | -0.39 |
