# Supplementary material for: Decontamination of N95 and surgical masks using a treatment based on a continuous gas phase-Advanced Oxidation Process
Source: PLoS One. 2021 Mar 18;16(3):e0248487. doi: 10.1371/journal.pone.0248487 (PMC7971510; doi:10.1371/journal.pone.0248487)
Supplement: S3 Table — (DOCX) [file pone.0248487.s008.docx]

Table S3: TCID_50_/ml of human coronavirus E299 inoculated onto mask sections then treated with gas phase Advanced Oxidation Process.

| Sample | Non-treated | gAOP treated |
| --- | --- | --- |
|  |  |  |
| 1 | >2 x 10^8^ | Not Detected |
| 2 | >2 x 10^8^ | Not Detected |
| 3 | >2 x 10^8^ | Not Detected |
| 4 | >2 x 10^8^ | Not Detected |
| Average | >2 x 10^8^ | 0 |
