# Supplementary material for: Decontamination of N95 and surgical masks using a treatment based on a continuous gas phase-Advanced Oxidation Process
Source: PLoS One. 2021 Mar 18;16(3):e0248487. doi: 10.1371/journal.pone.0248487 (PMC7971510; doi:10.1371/journal.pone.0248487)
Supplement: S4 Table — (DOCX) [file pone.0248487.s009.docx]

Table S4: Normalized cell viability before and after gas phase Advanced Oxidation Process.

|  | **Relative Light Units (RLU)** | |
| --- | --- | --- |
| **Mask Sample** | **Non-treated** | **gAOP Treated** |
| 1 | 0.910 | 0.946 |
| 2 | 0.835 | 0.939 |
| 3 | 0.897 | 0.916 |
| 4 | 0.824 | 0.956 |
| Average | 0.867 | 0.939 |
