# Supplementary material for: Decontamination of N95 and surgical masks using a treatment based on a continuous gas phase-Advanced Oxidation Process
Source: PLoS One. 2021 Mar 18;16(3):e0248487. doi: 10.1371/journal.pone.0248487 (PMC7971510; doi:10.1371/journal.pone.0248487)
Supplement: S5 Table — (DOCX) [file pone.0248487.s010.docx]

Table S5: Evaluation of filter penetration and pressure drop of N95 masks passed through the gas phase-Advanced Oxidation Process decontamination treatment 10 or 20 times compared to non-treated controls.

| **Treatment** | **Flow Rate**  **(l/min)** | **Pressure Drop (mm Hg)** | **Filter Penetration (%)** |
| --- | --- | --- | --- |
| Control | 84.7 | 7.2 | 0.17 |
| Control | 84.8 | 7.7 | 0.093 |
| Control | 84.9 | 7.7 | 0.183 |
| 10 Passes through Clean Flow | 84.9 | 6.9 | 0.292 |
| 10 Passes through Clean Flow | 84.8 | 8 | 0.122 |
| 10 Passes through Clean Flow | 84.9 | 7.5 | 0.14 |
| 10 Passes through Clean Flow | 84.9 | 7.9 | 0.096 |
| 10 Passes through Clean Flow | 84.9 | 7.3 | 0.184 |
| 10 Passes through Clean Flow | 84.8 | 7.2 | 0.198 |
| 10 Passes through Clean Flow | 84.9 | 7.8 | 0.08 |
| 10 Passes through Clean Flow | 84.9 | 7.5 | 0.338 |
| 10 Passes through Clean Flow | 85.2 | 7.9 | 0.132 |
| 10 Passes through Clean Flow | 85.3 | 7.7 | 0.092 |
| 20 Passes through Clean Flow | 85.2 | 7.7 | 0.096 |
| 20 Passes through Clean Flow | 85.1 | 7.5 | 0.182 |
| 20 Passes through Clean Flow | 85.4 | 7.7 | 0.26 |
| 20 Passes through Clean Flow | 85.4 | 7.9 | 0.211 |
| 20 Passes through Clean Flow | 84.9 | 7.6 | 0.185 |
| 20 Passes through Clean Flow | 84.7 | 7.7 | 0.174 |
| 20 Passes through Clean Flow | 84.6 | 7.7 | 0.145 |
| 20 Passes through Clean Flow | 84.9 | 8 | 0.088 |
| 20 Passes through Clean Flow | 85.1 | 7.2 | 0.192 |
| 20 Passes through Clean Flow | 85.0 | 7.8 | 0.123 |
