# Supplementary material for: Decontamination of N95 and surgical masks using a treatment based on a continuous gas phase-Advanced Oxidation Process
Source: PLoS One. 2021 Mar 18;16(3):e0248487. doi: 10.1371/journal.pone.0248487 (PMC7971510; doi:10.1371/journal.pone.0248487)
Supplement: S6 Table — (DOCX) [file pone.0248487.s011.docx]

Table S6: Evaluation of headband integrity of N95 masks passed through the gas phase-Advanced Oxidation Process decontamination treatment 10 or 20 times compared to non-treated controls.

| **Treatment** | **Band Location** | **Headband Force 3^rd^ Cycle 50%** |
| --- | --- | --- |
| Control | Upper | 0.40 |
| Control | Lower | 0.42 |
| Control | Upper | 0.37 |
| Control | Lower | 0.42 |
| Control | Upper | 0.38 |
| Control | Lower | 0.42 |
| Control | Upper | 0.39 |
| Control | Lower | 0.44 |
| Control | Upper | 0.39 |
| Control | Lower | 0.43 |
| 20 Passes through Clean Flow | Upper | 0.40 |
| 20 Passes through Clean Flow | Lower | 0.41 |
| 20 Passes through Clean Flow | Upper | 0.39 |
| 20 Passes through Clean Flow | Lower | 0.43 |
| 20 Passes through Clean Flow | Upper | 0.39 |
| 20 Passes through Clean Flow | Lower | 0.41 |
| 20 Passes through Clean Flow | Upper | 0.40 |
| 20 Passes through Clean Flow | Lower | 0.42 |
| 20 Passes through Clean Flow | Upper | 0.40 |
| 20 Passes through Clean Flow | Lower | 0.43 |
| 20 Passes through Clean Flow | Upper | 0.38 |
| 20 Passes through Clean Flow | Lower | 0.42 |
| 20 Passes through Clean Flow | Upper | 0.38 |
| 20 Passes through Clean Flow | Lower | 0.44 |
| 20 Passes through Clean Flow | Upper | 0.40 |
| 20 Passes through Clean Flow | Lower | 0.41 |
| 20 Passes through Clean Flow | Upper | 0.40 |
